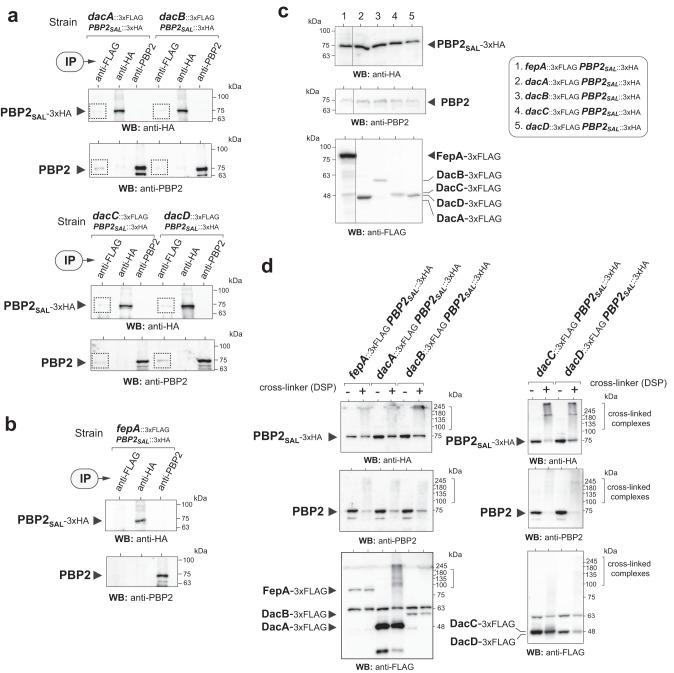# Author Correction: Evidence of two differentially regulated elongasomes in *Salmonella*

**DOI:** 10.1038/s42003-023-05381-1

**Published:** 2023-09-28

**Authors:** Sónia Castanheira, Francisco García-del Portillo

**Affiliations:** Laboratory of Intracellular Bacterial Pathogens, National Centre for Biotechnology (CNB)-CSIC, Darwin 3, 28049 Madrid, Spain

**Keywords:** Bacterial physiology, Cellular microbiology

Correction to: *Communications Biology* 10.1038/s42003-023-05308-w, published online 09 September 2023.

In this article, Fig. 6d contained an error related to the position of the (−) and (+) symbols on top of the Western blots; the figure should have appeared as shown below. The original article has been corrected.