# Supplementary material for: Evidence of two differentially regulated elongasomes in Salmonella
Source: Commun Biol. 2023 Sep 9;6:923. doi: 10.1038/s42003-023-05308-w (PMC10492807; doi:10.1038/s42003-023-05308-w)
Supplement: Supplementary file 2 — Supplementary Information [file 42003_2023_5308_MOESM2_ESM.pdf]

Supplementary Information

Evidence of two differentially regulated elongasomes in *Salmonella*

Sónia Castanheira and Francisco García-del Portillo

This PDF includes:

Supplementary **Figures 1 to 22**.

Supplementary **Table 1.**

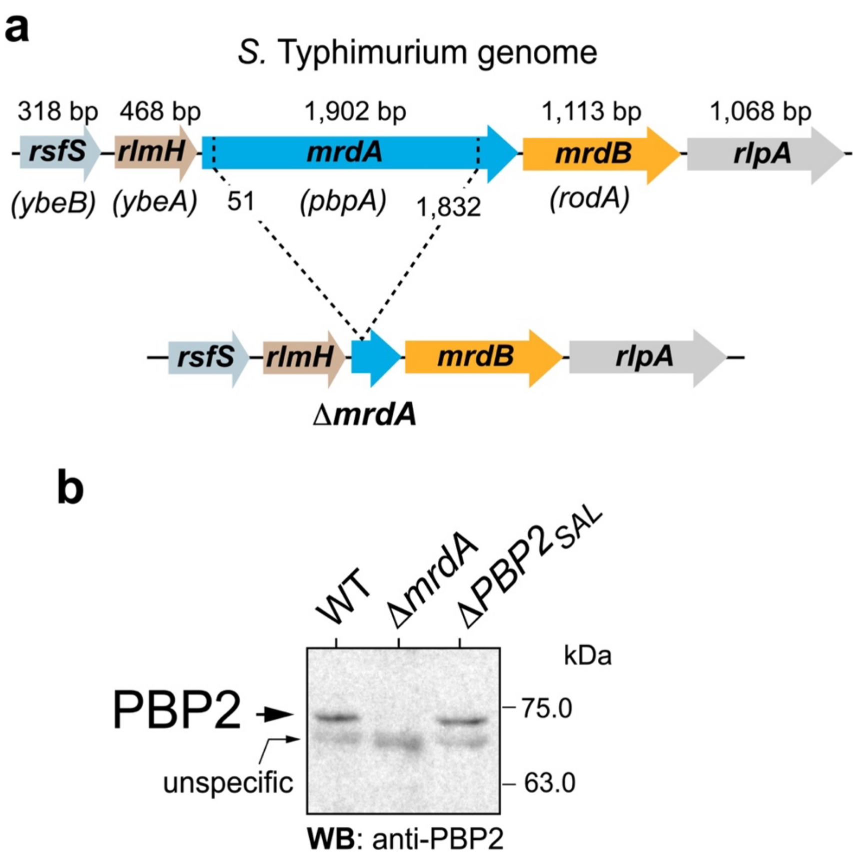

Supplementary Fig. 1. Construction of a *S.* Typhimurium Δ*mrdA* mutant defective in PBP2. **(a)** Region of the *S.* Typhimurium genome in which the gene encoding PBP2, *mrdA*, maps. Downstream is *mrdB*, the gene encoding the SEDS protein RodA, which contributes to PG synthesis with GTase activity in concert with the TPase activity of PBP2. Dashed lines indicate the region that was deleted in the Δ*mrdA* mutant encompassing nucleotide 51 to 1,832 of the coding sequence. (**b)** Western blot analysis with anti-PBP2 antibody showing the deficiency in PBP2 in the Δ*mrdA* mutant. Positions and sizes of molecular weight markers (in kDa) are indicated. The position of an unspecific protein detected with the antibody is also shown (*n*=2 biological).

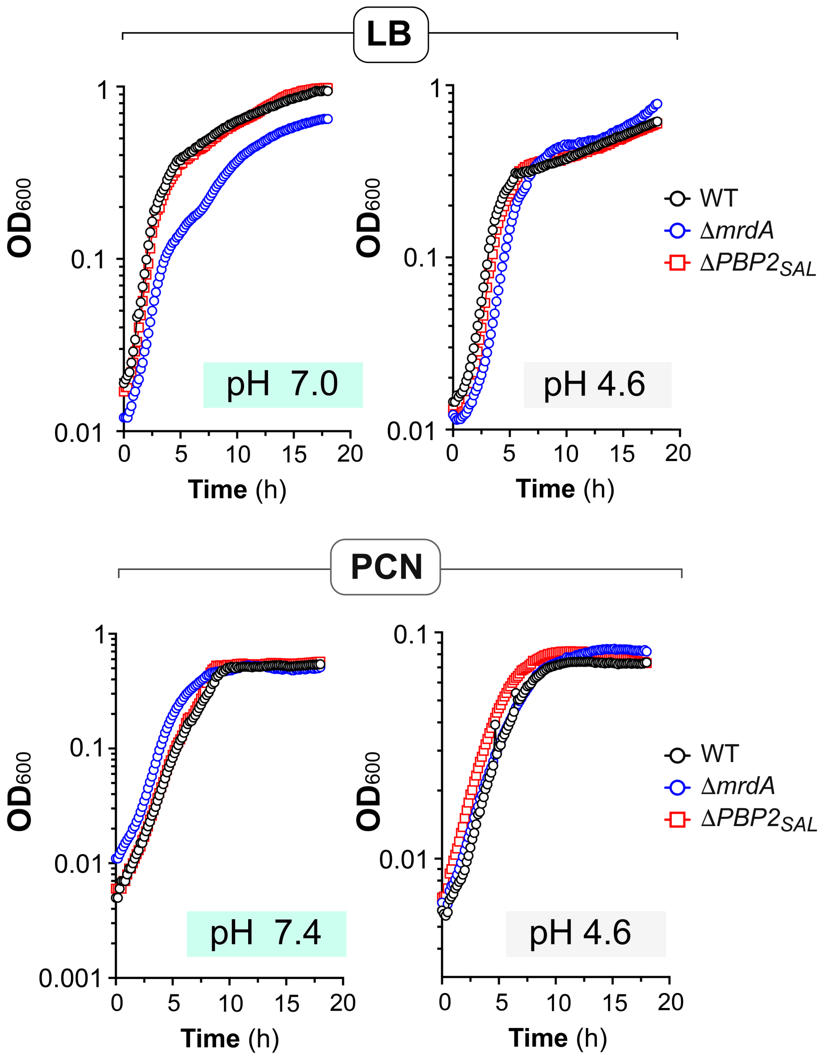

Supplementary Fig. 2. **Growth kinetics of *S.* Typhimurium isogenic mutants defective in PBP2 or PBP2$_{SAL}$ growing in nutrient rich (LB) or minimal (PCN) media at neutral or acidic pH**. The growth of these strains under the indicated conditions was monitored in a Tecan Spark microplate reader at 37ºC for 20 h. Note that the loss of PBP2 (Δ*mrdA* mutant) results in progressive decrease of the growth rate in LB medium at neutral pH, condition in which cells undergo drastic morphological changes as giant rounded cells (Fig. 1a, main manuscript). This Δ*mrdA* mutant however increases in mass similarly to wild type bacteria at neutral pH in PCN medium. Under this condition, cells acquire rounded morphology but do not show signs of blockage in cell division (Fig. 1a, main manuscript). Data are from a representative experiment (*n*=3 biological).

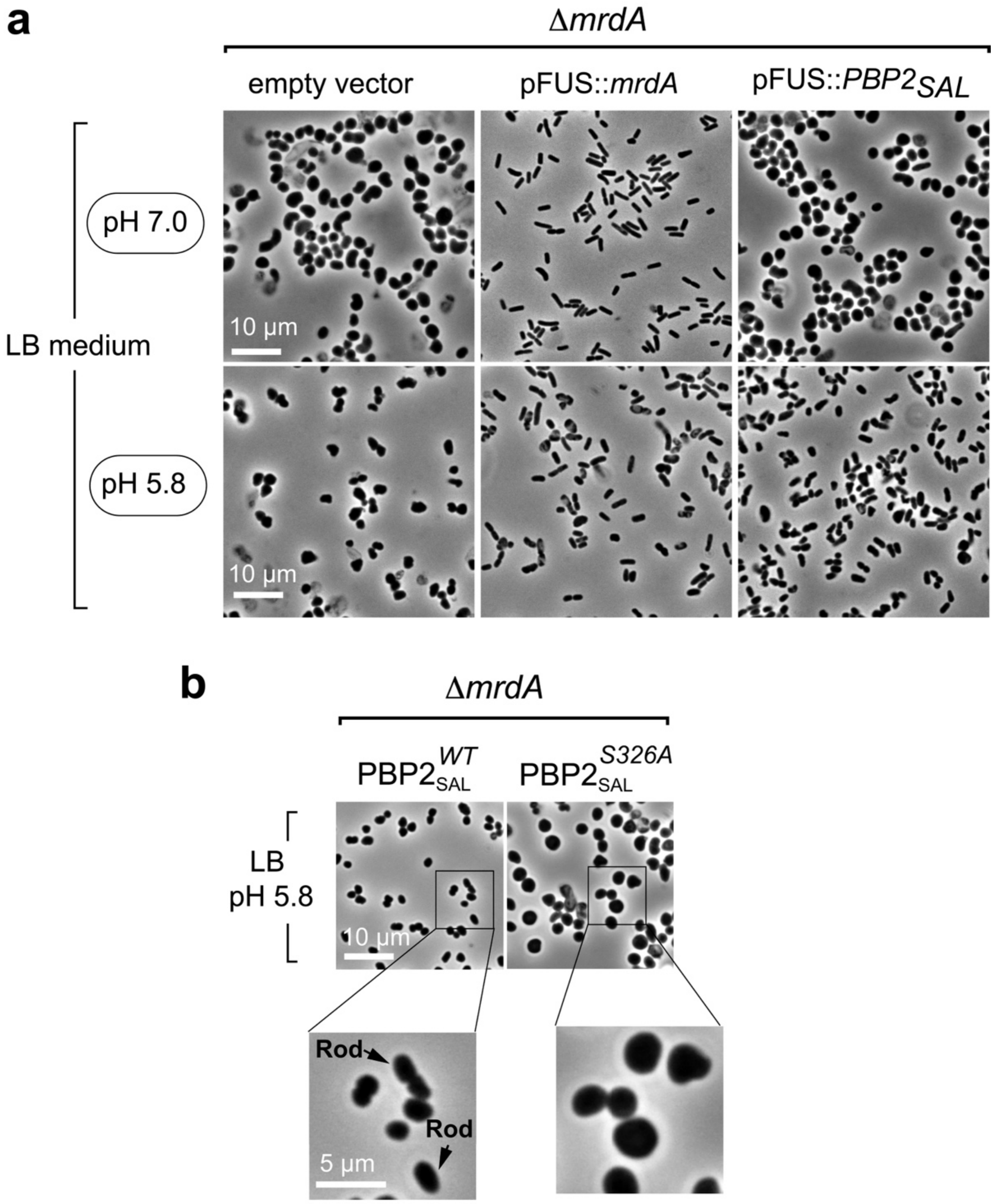

Supplementary Fig. 3. The serine326 (S326) of PBP2$_{SAL}$ is required for catalysis. (a) Morphology of Δ*mrdA* cells bearing plasmids with the pFUS backbone for ectopic expression of wild-type PBP2 or PBP2$_{SAL}$. A strain bearing the empty vector (pFUS) was used as control. Unlike PBP2, expression of PBP2$_{SAL}$ restores rod shape only in acidic pH (5.8) (*n*=2 biological). Scale bar, 10 µm. **(b)** Expression in Δ*mrdA* cells of a PBP2$_{SAL}$ variant with a S326A mutation does not generate rod shape morphology. This result demonstrates the strict requirement for catalysis of the S326 residue and that PBP2$_{SAL}$ can promote cell elongation in acidic pH in the absence of PBP2 (*n*=2 biological). Scale bars, 10 µm (low magnification) and 5 µm (high magnification).

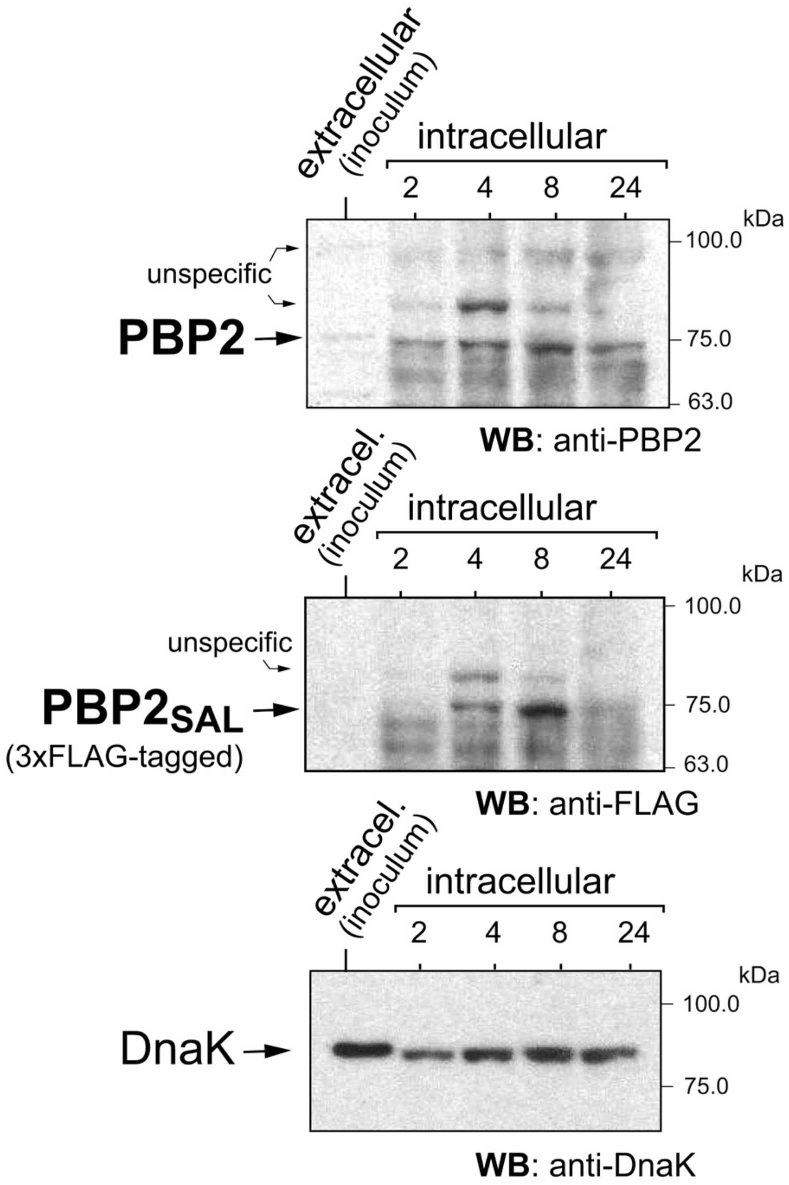

Supplementary Fig. 4. PBP2$_{SAL}$ expression is upregulated by *S.* Typhimurium inside fibroblasts. Western blot analyses showing relative levels of PBP2, PBP2$_{SAL}$ and DnaK in total protein extracts prepared from extracellular bacteria (input, inoculum) and intracellular bacteria at the indicated post-infection times (2, 4, 8, and 24 h). For this assay, wild type bacteria bearing the PBP2$_{SAL}$::3xFLAG epitope were grown overnight in LB pH 7.0 and used to infect cultured NRK-49F rat fibroblasts (*n*=2 biological). Positions and sizes of molecular weight markers (in kDa) are indicated. The position of unspecific protein detected with the antibodies is also shown.

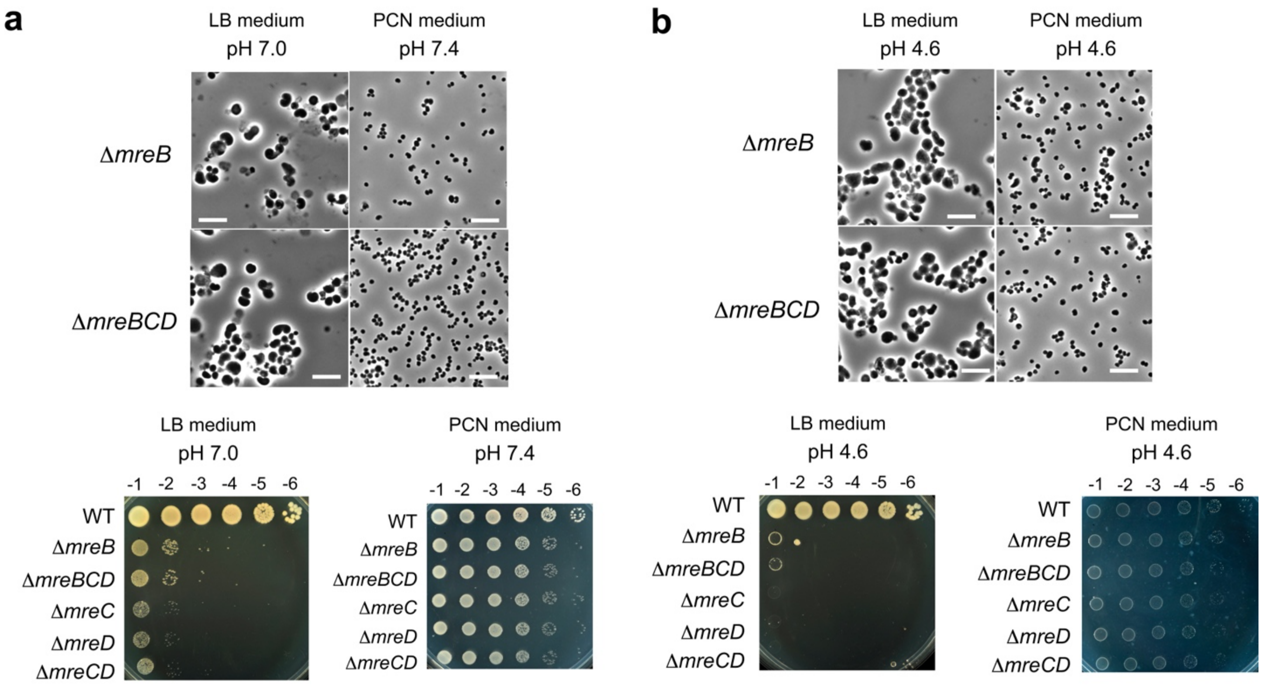

Supplementary Fig. 5. MreB, MreC and MreD are essential in *S.* Typhimurium to generate rod shape in both neutral and acidic pH. (a) Morphology and survival of isogenic mutants Δ*mreB* and Δ*mreBCD* in nutrient-rich (LB) and nutrient-poor (PCN) media at neutral pH (7.0 in LB, 7.4 in PCN). Scale bar, 10 µm. Morphology of the Δ*mreC* and Δ*mreD* single mutants was indistinguishable from that of Δ*mreB* and Δ*mreBCD* isogenic strains. The survival assay involving spotting of serial dilutions is depicted for all strains (*n*=3 biological). **(b)** Morphology and survival of the same set of strains described in **(a)** but grown in LB or PCN media in acidic pH (4.6). Scale bar, 10 µm. Morphology of the Δ*mreC* and Δ*mreD* single mutants in acidic pH was also indistinguishable from that of Δ*mreB* and Δ*mreBCD* isogenic strains (*n*=3 biological). Scale bar, 10 µm.

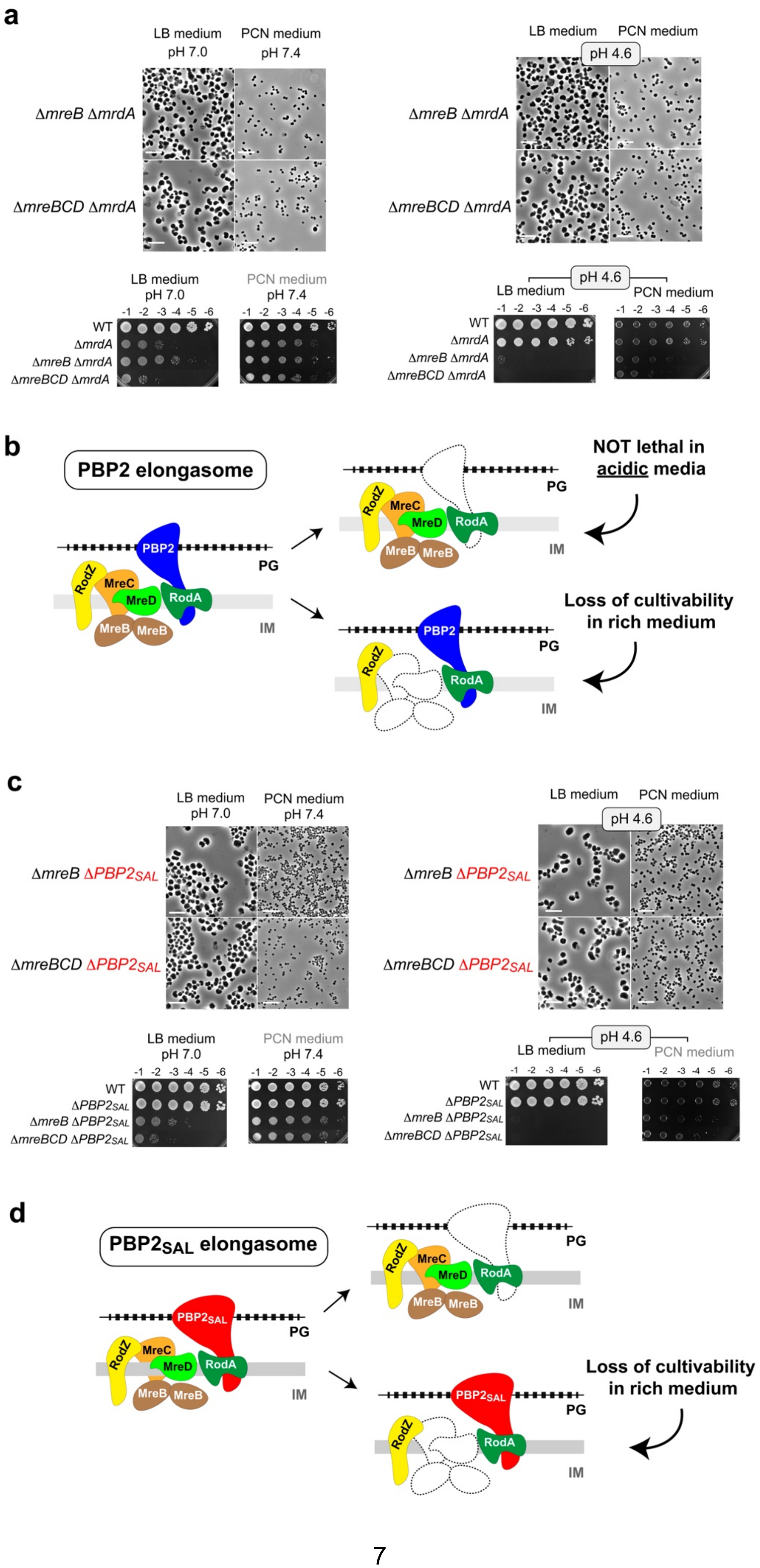

a
LB medium pH 7.0
PCN medium pH 7.4
ΔmreB ΔmrdA
ΔmreBCD ΔmrdA
LB medium
PCN medium
pH 4.6
WT
ΔmrdA
ΔmreB ΔmrdA
ΔmreBCD ΔmrdA
-1 -2 -3 -4 -5 -6
b
PBP2 elongasome
PBP2
PG
IM
RodZ
MreC
MreD
RodA
MreB
NOT lethal in acidic media
Loss of cultivability in rich medium
c
ΔmreB ΔPBP2SAL
ΔmreBCD ΔPBP2SAL
WT
ΔPBP2SAL
d
PBP2SAL elongasome
PBP2SAL

Supplementary Fig. 6. Unlike in neutral pH, the loss of PBP2 in acidic pH does not affect survival in the presence of MreB, MreC and MreD. (a) Morphology and viability of isogenic Δ*mreB*Δ*mrdA* and Δ*mreBCD*Δ*mrdA* cells grown in the indicated nutrient media and pH (*n*=3 biological). Scale bar, 10 µm. (**b)** Scheme of the essentiality of the different components in the PBP2 elongasome. The loss of PBP2 or MreBCD affect cultivability in neutral pH, however PBP2 is dispensable in acidic pH. **(c)** Morphology and viability of isogenic Δ*mreB*Δ$PBP2_{SAL}$ and Δ*mreBCD*Δ$PBP2_{SAL}$ cells grown in the indicated nutrient media and pH (*n*=3 biological). Scale bar, 10 µm. **(d)** Scheme of the essentiality of the different components in the $PBP2_{SAL}$ elongasome, active in acidic pH conditions. Loss of cultivability is observed only upon loss of the MreBCD components.

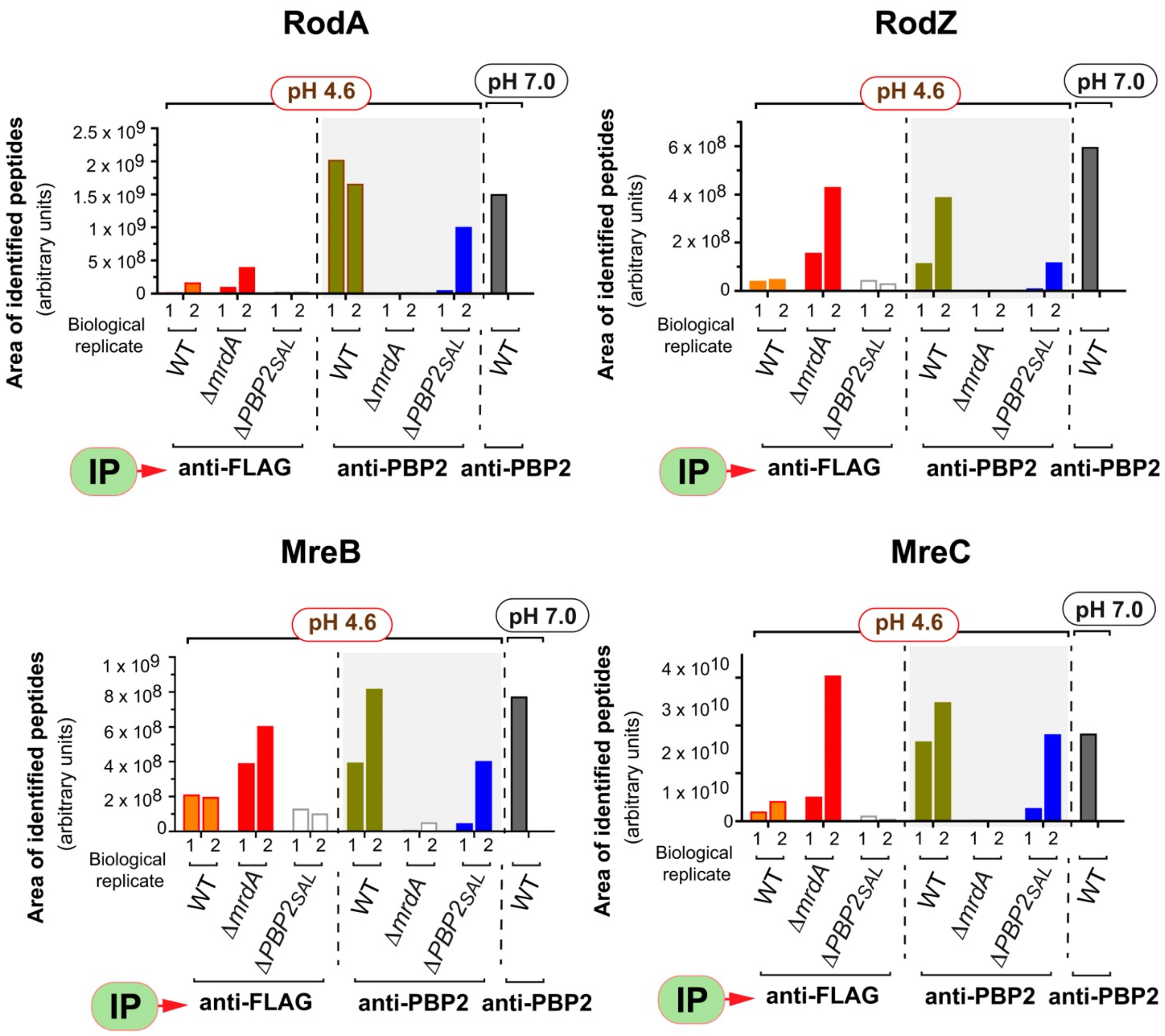

Supplementary Fig. 7. Elongasome proteins identified by mass spectrometry as components of the PBP2- and PBP2$_{SAL}$-morphogenetic complexes. The sum of areas corresponding to distinct peptides identified for each of the indicated proteins is shown for the complexes immunoprecipitated (IP) with either anti-FLAG (PBP2$_{SAL\text{-}3xFLAG}$) or anti-PBP2 antibodies in the strains shown (*n*=2 biological, except sample of neutral pH, *n*=1). See complete set of data for individual peptides in the "Supplementary Data" file. The figures also refer to the distinct pH used. The elongasomes immunoprecipitated from wild type (WT), Δ*mrdA* and Δ*PBP2*$_{SAL}$ strains were pull-down from bacteria grown in LB pH 4.6. For WT cells, an additional sample was analyzed from bacteria grown at pH 7.0.

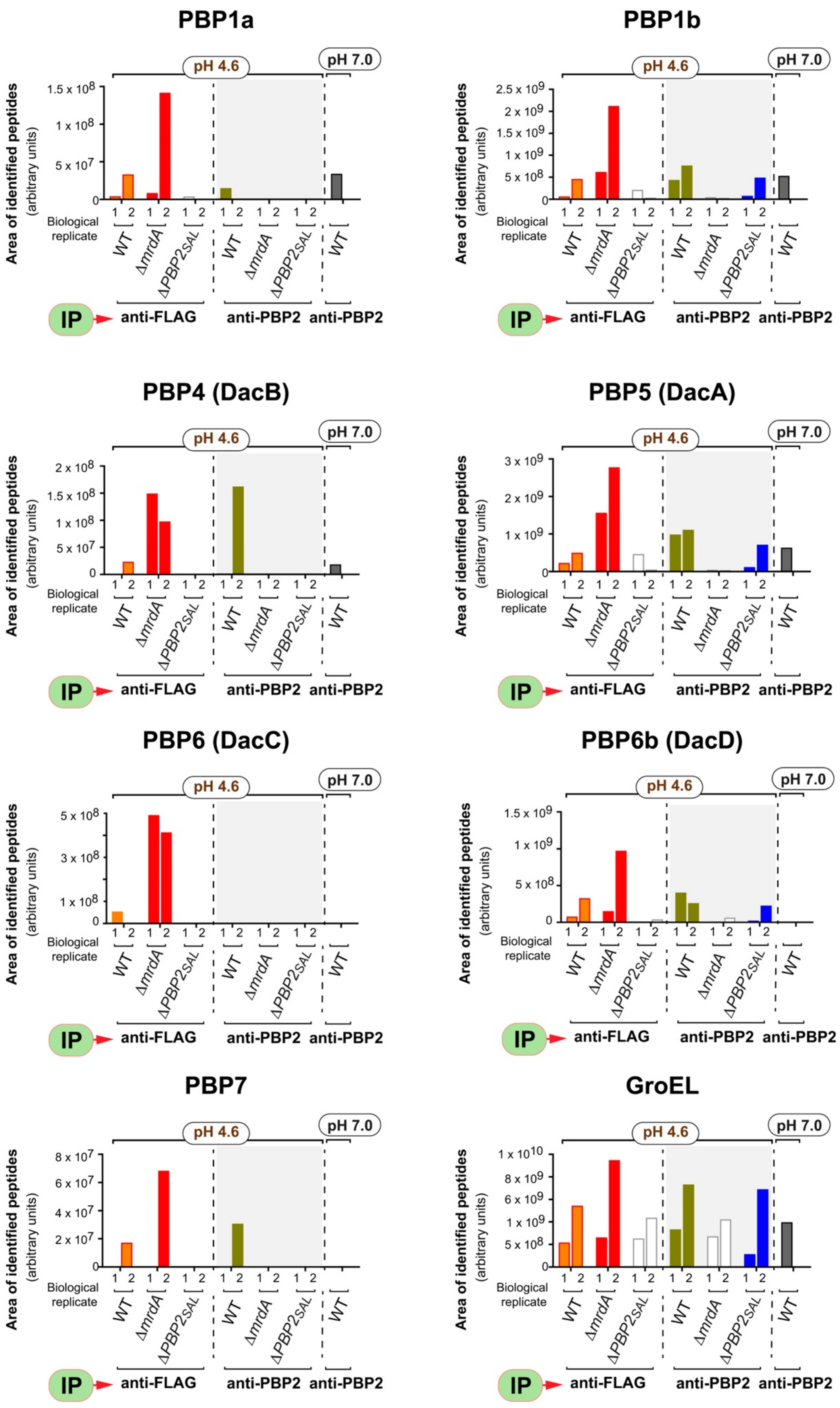
PBP1a
PBP1b
PBP4 (DacB)
PBP5 (DacA)
PBP6 (DacC)
PBP6b (DacD)
PBP7
GroEL
pH 4.6
pH 7.0
Area of identified peptides
(arbitrary units)
Biological replicate
1 2
WT
ΔmrdA
ΔPBP2SAL
IP
anti-FLAG
anti-PBP2
anti-PBP2

Supplementary Fig. 8. PBPs identified by mass spectrometry as components of the PBP2- and PBP2$_{SAL}$-elongasomes. The sum of areas corresponding to peptides identified for each of the indicated proteins is shown for the complexes immunoprecipitated (IP) with either anti-FLAG (PBP2$_{SAL\text{-}3xFLAG}$) or anti-PBP2 antibodies in the isogenic strains shown (*n*=2 biological, except sample of neutral pH, *n*=1). See complete set of data for individual peptides in the "Supplementary Data" file. Growth conditions were LB medium pH 4.6 for wild type (WT), Δ*mrdA* and Δ*PBP2$_{SAL}$* strains. For WT bacteria, an additional sample was analyzed at pH 7.0. GroEL was analyzed as unrelated and unspecific control protein detected at similar level in all samples.

a

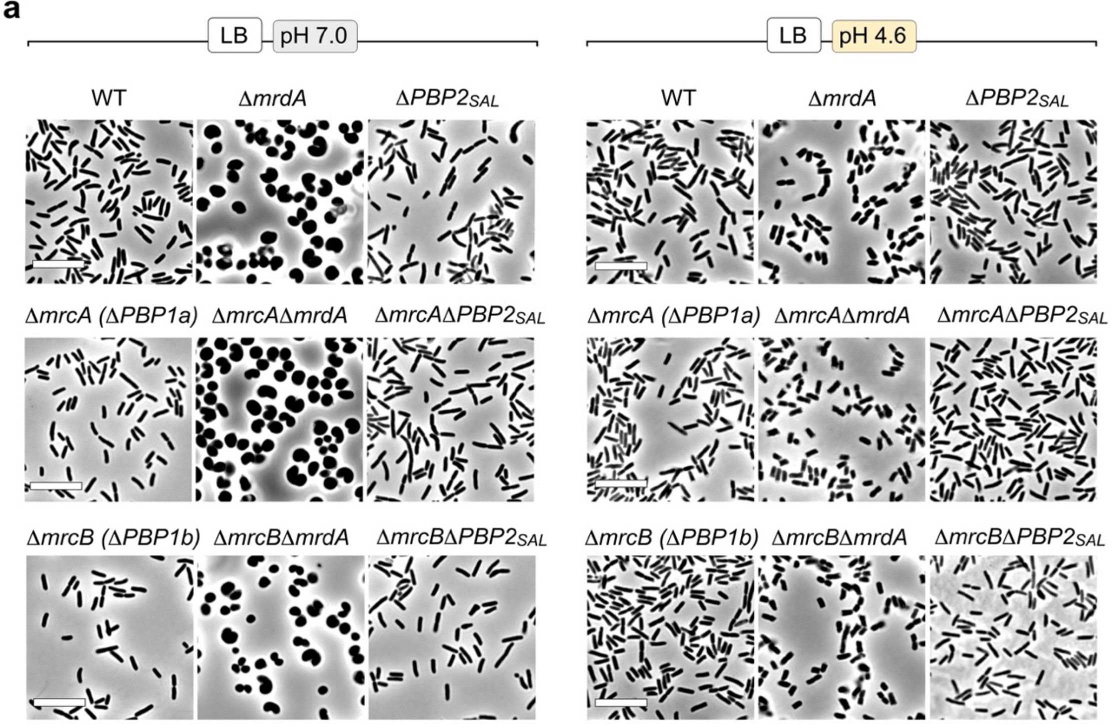

b

	pH 7.0		Number of cells (*n*)	OD_{600} value	pH 4.6		Number of cells (*n*)	OD_{600} value
	**Length (L)** (µm)	**Width (W)** (µm)			**L** (µm)	**W** (µm)		
WT	3.11 ± 0.84	0.76 ± 0.08	765	0.45	3.13 ± 0.83	0.86 ± 0.11	373	0.45
Δ*mrdA*	(giant cells)	(giant cells)	--	0.23	2.45 ± 0.66	1.05 ± 0.16	539	0.32
Δ*PBP2*$_{SAL}$	3.19 ± 0.95	0.75 ± 0.10	434	0.44	2.90 ± 0.79	0.73 ± 0.11	769	0.47
Δ*mrcA* (Δ*PBP1a*)	2.90 ± 0.89	0.77 ± 0.09	571	0.48	2.87± 0.74	0.73 ± 0.11	796	0.46
Δ*mrcA*Δ*mrdA*	(giant cells)	(giant cells)	--	0.20	2.48 ± 0.67	0.99 ± 0.13	662	0.29
Δ*mrcA*Δ*PBP2*$_{SAL}$	3.30 ± 0.99	0.78 ± 0.09	467	0.40	2.88 ± 0.78	0.71 ± 0.09	941	0.47
Δ*mrcB* (Δ*PBP1b*)	3.24 ± 0.88	0.77 ± 0.10	386	0.43	3.00 ± 0.79	0.76 ± 0.11	662	0.42
Δ*mrcB*Δ*mrdA*	(giant cells)	(giant cells)	--	0.24	2.25 ± 0.57	0.99 ± 0.15	606	0.28
Δ*mrcB*Δ*PBP2*$_{SAL}$	3.45 ± 0.96	0.74 ± 0.09	632	0.41	2.88 ± 0.76	0.72 ± 0.11	531	0.43

Supplementary Fig. 9. The morphological parameters of *S.* Typhimurium mutants lacking PBP2 or PBP2$_{SAL}$ growing in LB are not altered by the loss of PBP1a or PBP1b. **(a)** Images of the indicated mutants. Bacteria were collected in the exponential phase at neutral and acidic pH. WT, wild type. Scale bar, 10 µm. (**b**) Average length and width determined with ObjectJ (https://sils.fnwi.uva.nl/bcb/objectj/) for the listed isogenic mutants. Indicated are the number of cells that were monitored and the OD_{600} value at which bacteria were collected. Mutants lacking PBP2 exhibit at neutral pH aberrant morphologies as giant rounded cells with gross differences in length and width, reason for which they were not examined with ObjectJ. Data are from a representative experiment (*n*=2 biological).

a

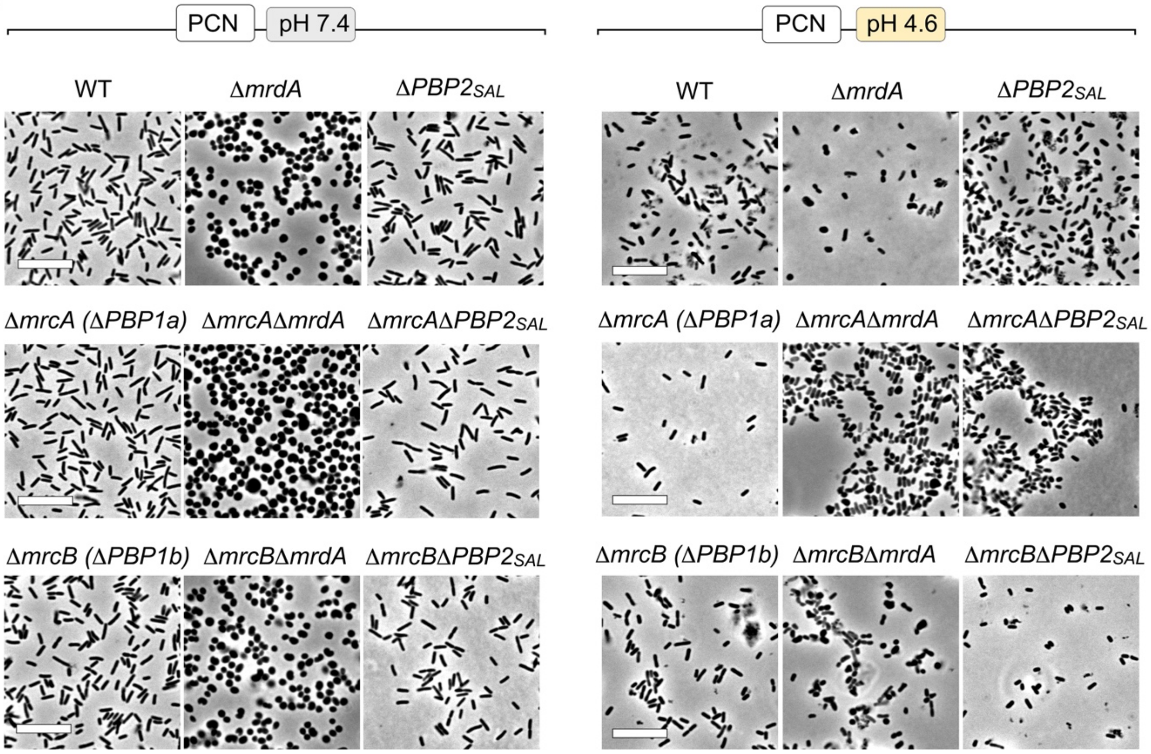

b

	**pH 7.4**		Number of cells (***n***)	OD_{600} value	**pH 4.6**		Number of cells (***n***)	OD_{600} value
	**Length (L)** (μm)	**Width (W)** (μm)			**L** (μm)	**W** (μm)		
WT	2.49 ± 0.58	0.65 ± 0.15	637	0.24	2.12 ± 0.50	0.72 ± 0.14	237	0.06
Δ*mrdA*	1.63 ± 0.31	1.22 ± 0.14	547	0.26	1.75 ± 0.34	0.91 ± 0.19	184	0.04
Δ*PBP2*$_{SAL}$	2.48 ± 0.60	0.67 ± 0.17	498	0.26	1.83 ± 0.37	0.83 ± 0.12	323	0.06
Δ*mrcA* (Δ*PBP1a*)	2.52 ± 0.61	0.65 ± 0.14	721	0.25	2.18 ± 0.54	0.72 ± 0.09	135	0.06
Δ*mrcA*Δ*mrdA*	1.53 ± 0.28	1.15 ± 0.16	620	0.26	1.77 ± 0.30	0.87 ± 0.20	208	0.06
Δ*mrcA*Δ*PBP2*$_{SAL}$	2.58 ± 0.67	0.67 ± 0.16	433	0.23	1.85 ± 0.34	0.80 ± 0.15	143	0.06
Δ*mrcB* (Δ*PBP1b*)	2.25 ± 0.55	0.70 ± 0.16	619	0.26	2.15 ± 0.47	0.73 ± 0.16	185	0.05
Δ*mrcB*Δ*mrdA*	1.64 ± 0.36	1.21 ± 0.14	516	0.26	1.84 ± 0.37	0.85 ±0.18	189	0.05
Δ*mrcB*Δ*PBP2*$_{SAL}$	2.43 ± 0.57	0.69 ± 0.17	404	0.19	1.95 ± 0.34	0.73 ± 0.15	106	0.04

Supplementary Fig. 10. The morphological parameters of *S.* Typhimurium mutants defective in PBP2 or PBP2$_{SAL}$ growing in minimal PCN medium are not altered by the loss of PBP1a or PBP1b. **(a)** Images of the indicated mutants. Bacteria were collected in the exponential phase at neutral and acidic pH. WT, wild type. Scale bar, 10 μm. (**b**) Average length and width determined with ObjectJ (https://sils.fnwi.uva.nl/bcb/objectj/) for the listed isogenic mutants. Indicated are the number of cells that were monitored and the OD_{600} value at which bacteria were collected. Data are from a representative experiment (*n*=2 biological).

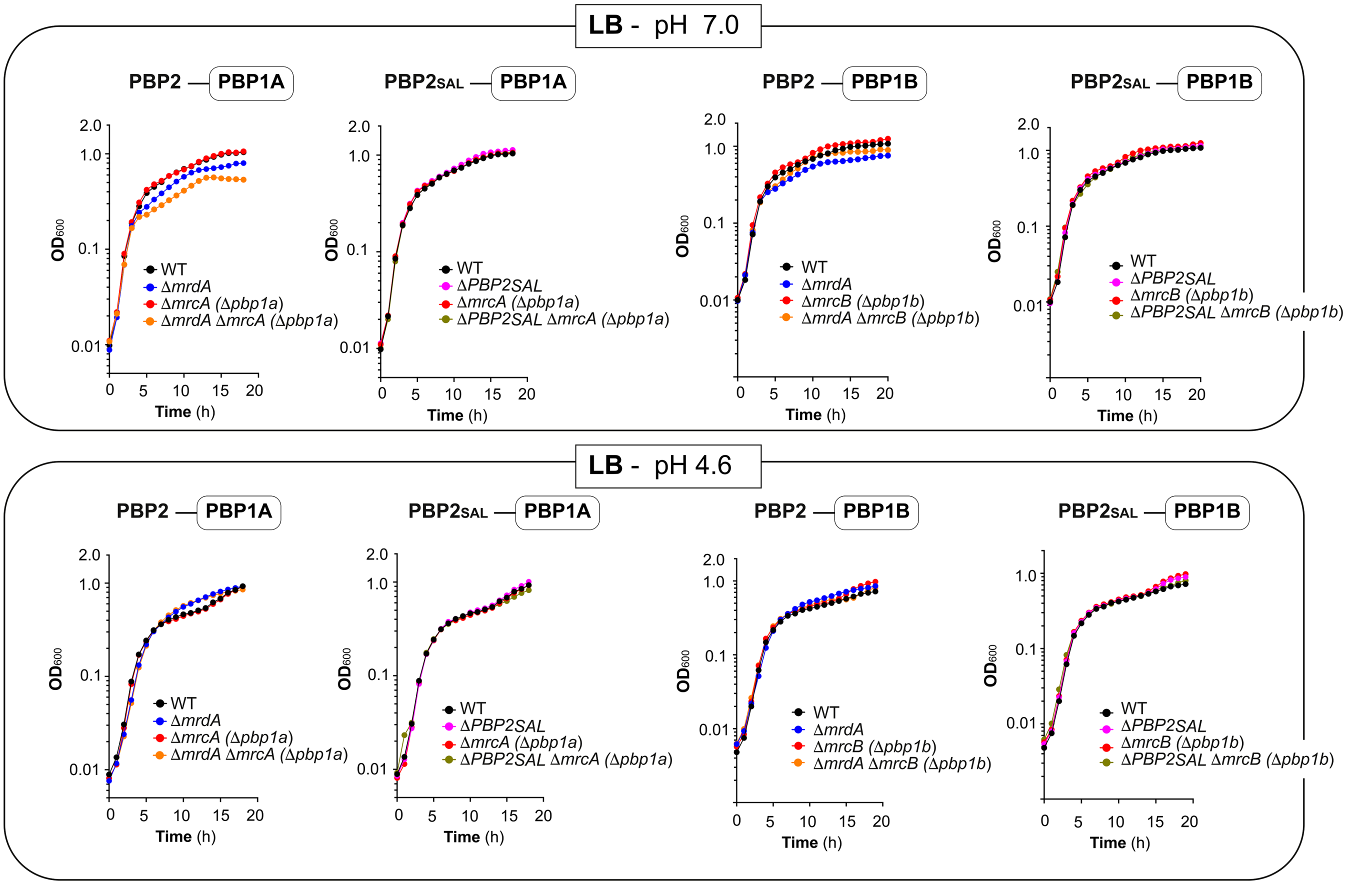

Supplementary Fig. 11. Growth curves of *S.* Typhimurium mutants defective in PBP2 or $\mathbf{PBP2_{SAL}}$ and the bifunctional PBP1a (MrcA) or PBP1b (MrcB) cultured in nutrient rich LB medium at neutral or acidic pH. Growth was monitored over time in a Tecan Spark microplate reader at 20 min intervals by measuring optical density at 600 nm wavelength. Note the lower final OD_{600} values in Δ*mrdA* mutants lacking PBP2 growing at pH 7.0, associated with the formation of large, rounded cells unable to divide (see Fig. 1a main manuscript). (*n*=2 biological).

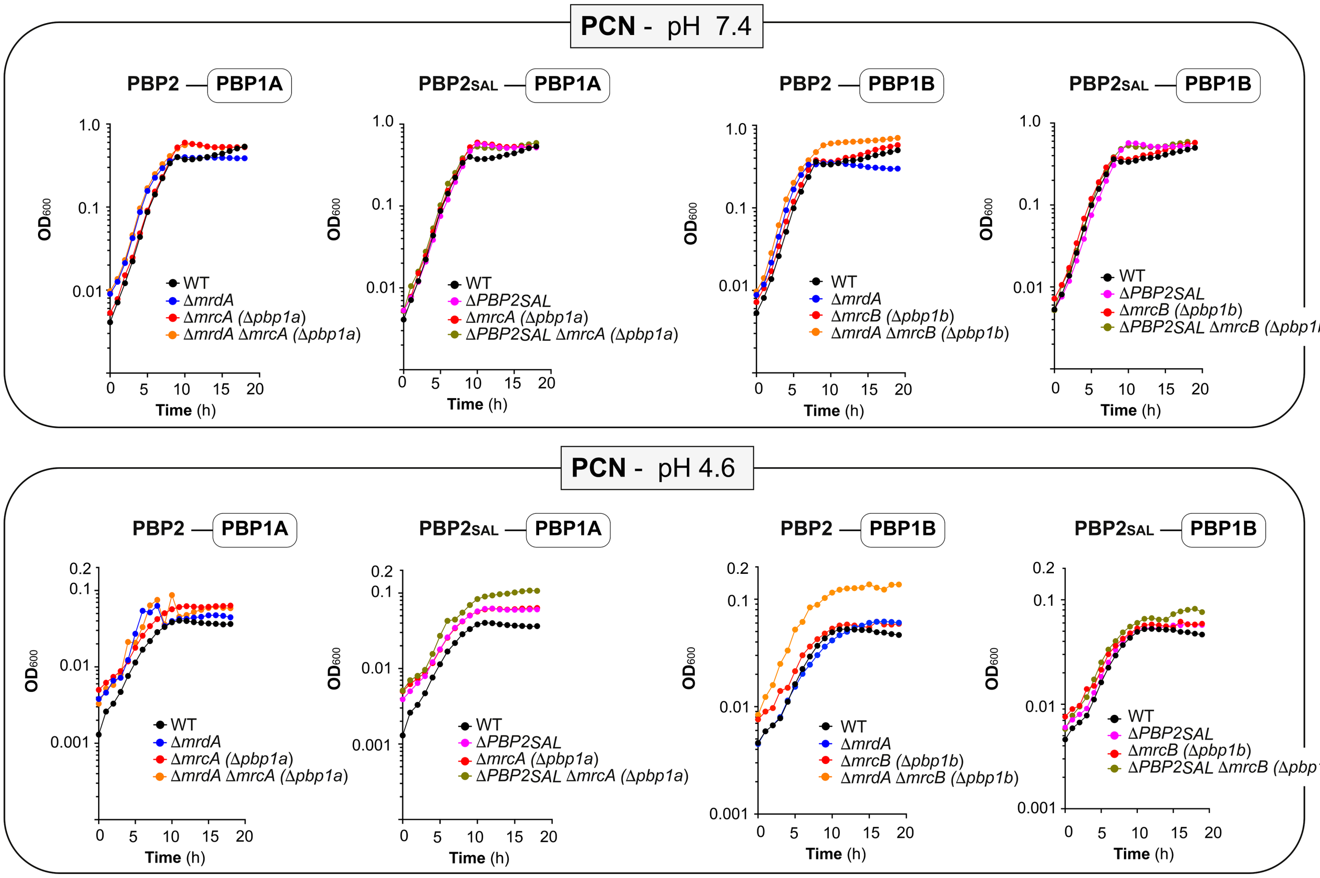

Supplementary Fig. 12. Growth curves of *S.* Typhimurium mutants defective in PBP2 or $PBP2_{SAL}$ and the bifunctional PBP1a (MrcA) or PBP1b (MrcB) cultured in minimal nutrient poor PCN medium at neutral or acidic pH. Growth was monitored over time in a Tecan Spark microplate reader at 20 min intervals by measuring optical density at 600 nm wavelength. Note the increase in final OD_{600} values for mutants lacking PBP1a growing at acidic pH in the absence of PBP2. A similar effect is observed for the mutant lacking PBP1a and $PBP2_{SAL}$. (*n*=2 biological).

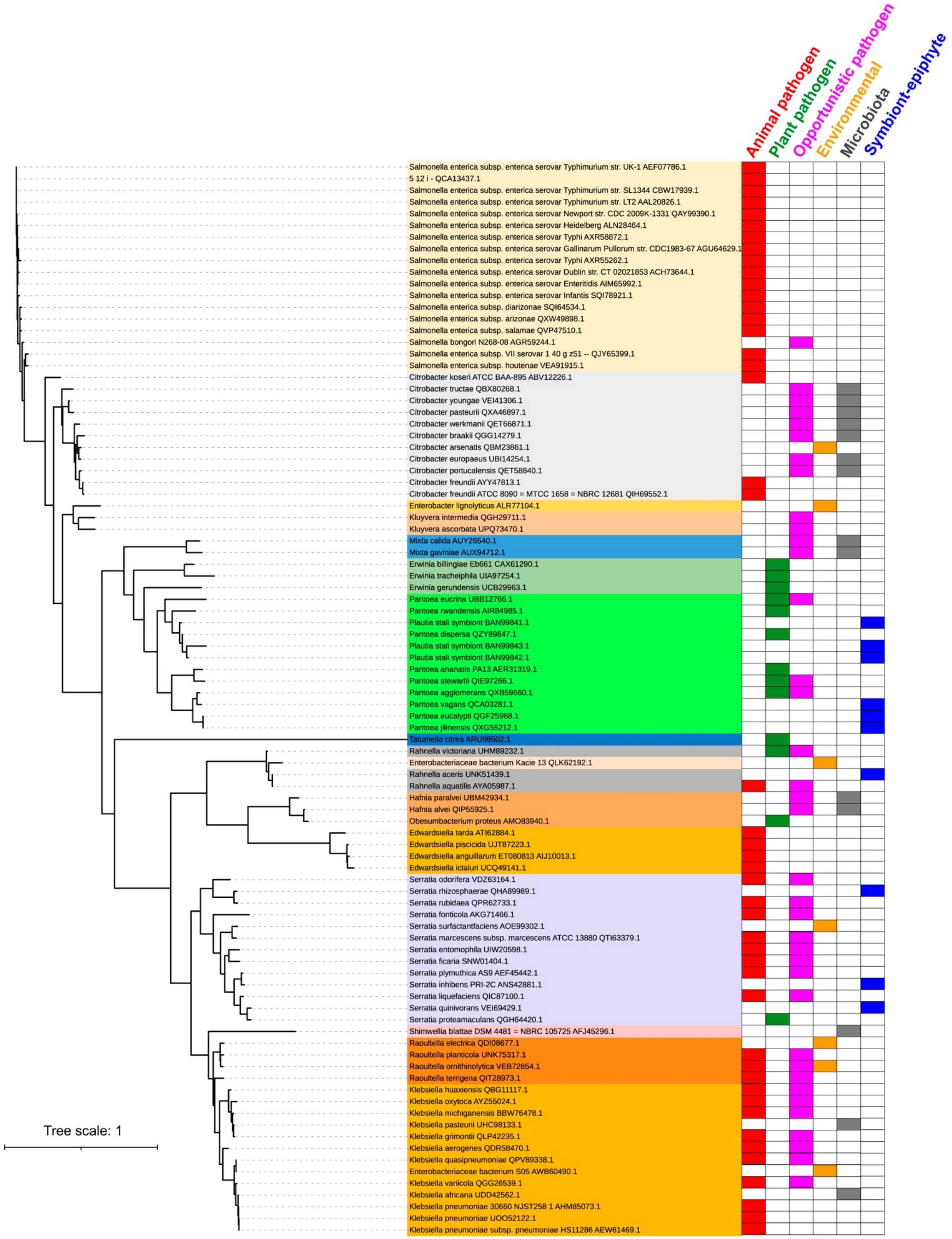
Animal pathogen
Plant pathogen
Opportunistic pathogen
Environmental
Microbiota
Symbiont-epiphyte
Salmonella enterica subsp. enterica serovar Typhimurium str. UK-1 AEF07786.1
5 12 i - QCA13437.1
Salmonella enterica subsp. enterica serovar Typhimurium str. SL1344 CBW17939.1
Salmonella enterica subsp. enterica serovar Typhimurium str. LT2 AAL20826.1
Salmonella enterica subsp. enterica serovar Newport str. CDC 2009K-1331 QAY99390.1
Salmonella enterica subsp. enterica serovar Heidelberg ALN28464.1
Salmonella enterica subsp. enterica serovar Typhi AXR58872.1
Salmonella enterica subsp. enterica serovar Gallinarum Pullorum str. CDC1983-67 AGU64629.1
Salmonella enterica subsp. enterica serovar Typhi AXR55262.1
Salmonella enterica subsp. enterica serovar Dublin str. CT 02021853 ACH73644.1
Salmonella enterica subsp. enterica serovar Enteritidis AIM65992.1
Salmonella enterica subsp. enterica serovar Infantis SQI78921.1
Salmonella enterica subsp. diarizonae SQI64534.1
Salmonella enterica subsp. arizonae QXW49898.1
Salmonella enterica subsp. salamae QVP47510.1
Salmonella bongori N268-08 AGR59244.1
Salmonella enterica subsp. VII serovar 1 40 g z51 -- QJY65399.1
Salmonella enterica subsp. houtenae VEA91915.1
Citrobacter koseri ATCC BAA-895 ABV12226.1
Citrobacter tructae QBX80268.1
Citrobacter youngae VEI41306.1
Citrobacter pasteurii QXA46897.1
Citrobacter werkmanii QET66871.1
Citrobacter braakii QGG14279.1
Citrobacter arsenatis QBM23861.1
Citrobacter europaeus UBI14254.1
Citrobacter portucalensis QET58840.1
Citrobacter freundii AYY47813.1
Citrobacter freundii ATCC 8090 = MTCC 1658 = NBRC 12681 QIH69552.1
Enterobacter lignolyticus ALR77104.1
Kluyvera intermedia QGH29711.1
Kluyvera ascorbata UPQ73470.1
Mixta calida AUY26540.1
Mixta gaviniae AUX94712.1
Erwinia billingiae Eb661 CAX61290.1
Erwinia tracheiphila UIA97254.1
Erwinia gerundensis UCB29963.1
Pantoea eucrina UBB12766.1
Pantoea rwandensis AIR84985.1
Plautia stali symbiont BAN99841.1
Pantoea dispersa QZY89847.1
Plautia stali symbiont BAN99843.1
Plautia stali symbiont BAN99842.1
Pantoea ananatis PA13 AER31319.1
Pantoea stewartii QIE97266.1
Pantoea agglomerans QXB59660.1
Pantoea vagans QCA03281.1
Pantoea eucalypti QGF25968.1
Pantoea jilinensis QXG55212.1
Tatumella citrea ARU98502.1
Rahnella victoriana UHM89232.1
Enterobacteriaceae bacterium Kacie 13 QLK62192.1
Rahnella aceris UNK51439.1
Rahnella aquatilis AYA05987.1
Hafnia paralvei UBM42934.1
Hafnia alvei QIP55925.1
Obesumbacterium proteus AMO83940.1
Edwardsiella tarda ATI62884.1
Edwardsiella piscicida UJT87223.1
Edwardsiella anguillarum ET080813 AIJ10013.1
Edwardsiella ictaluri UCQ49141.1
Serratia odorifera VDZ63164.1
Serratia rhizosphaerae QHA89989.1
Serratia rubidaea QPR62733.1
Serratia fonticola AKG71466.1
Serratia surfactantfaciens AOE99302.1
Serratia marcescens subsp. marcescens ATCC 13880 QTI63379.1
Serratia entomophila UIW20598.1
Serratia ficaria SNW01404.1
Serratia plymuthica AS9 AEF45442.1
Serratia inhibens PRI-2C ANS42881.1
Serratia liquefaciens QIC87100.1
Serratia quinivorans VEI69429.1
Serratia proteamaculans QGH64420.1
Shimwellia blattae DSM 4481 = NBRC 105725 AFJ45296.1
Raoultella electrica QDI08677.1
Raoultella planticola UNK75317.1
Raoultella ornithinolytica VEB72654.1
Raoultella terrigena QIT28973.1
Klebsiella huaxiensis QBG11117.1
Klebsiella oxytoca AYZ55024.1
Klebsiella michiganensis BBW76478.1
Klebsiella pasteurii UHC98133.1
Klebsiella grimontii QLP42235.1
Klebsiella aerogenes QDR58470.1
Klebsiella quasipneumoniae QPV89338.1
Enterobacteriaceae bacterium S05 AWB60490.1
Klebsiella variicola QGG26539.1
Klebsiella africana UDD42562.1
Klebsiella pneumoniae 30660 NJST258 1 AHM85073.1
Klebsiella pneumoniae UOO52122.1
Klebsiella pneumoniae subsp. pneumoniae HS11286 AEW61469.1
Tree scale: 1

Supplementary Fig. 13. Phylogenetic distribution of $PBP2_{SAL}$ orthologs in bacterial genera of the order *Enterobacterales*. The maximum-likehood phylogenetic tree was built by analyzing 325 representative and complete genomes of the order *Enterobacterales* with hits that BLASTp showed ≥ 60% identical and with ≥ 90% coverage versus $PBP2_{SAL}$ of *S.* Typhimurium SL1344 (Uniprot entry E1WGF1), used as query. See *Methods* for details. The columns on the right indicate the lifestyles of the distinct genera, which were classified according to the literature as pathogenic (animal or plant), opportunistic pathogen, environmental, microbiota (human or other animals), and symbiotic/epiphyte.

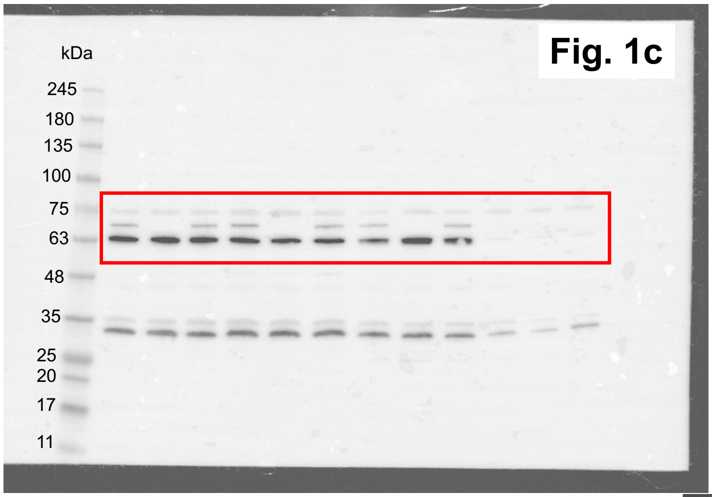

Fig. 1c. Upper image: Western blot anti-PBP2/PBP3

Fig. 1c. Middle image: Western blot anti-FLAG

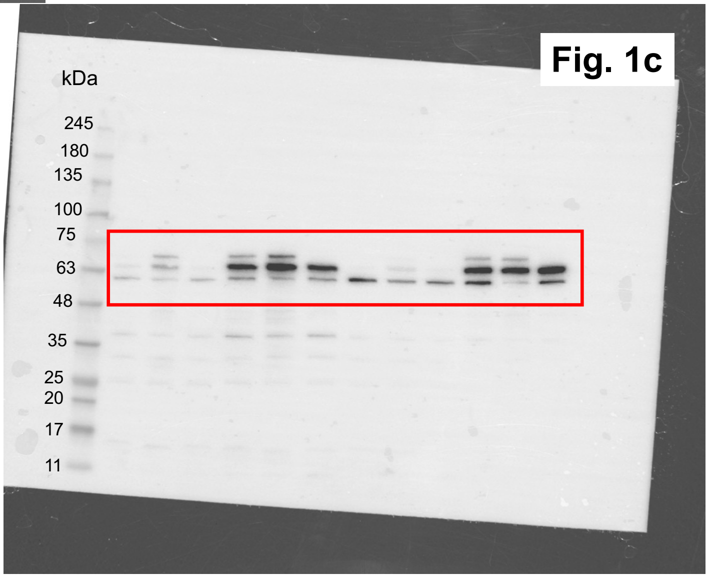

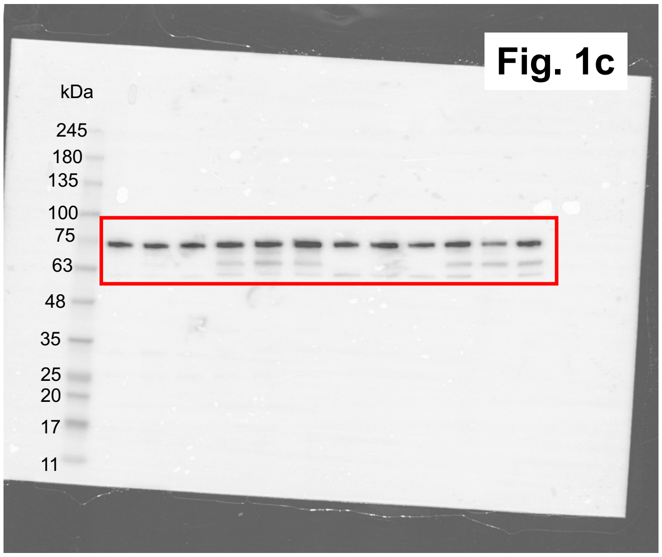

Fig. 1c. Lower image: Western blot anti-DnaK

Supplementary Fig. 14. Original uncropped Western blots corresponding to Figure 1c of manuscript. Red boxes denote the areas of the blots that were used to compose Figure 1c.

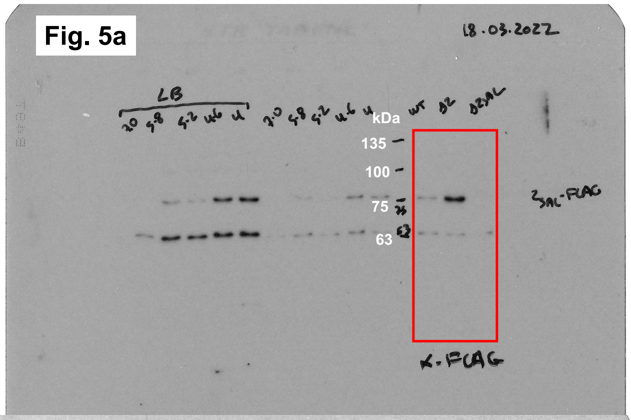

Fig. 5a. Upper image: Western anti-FLAG

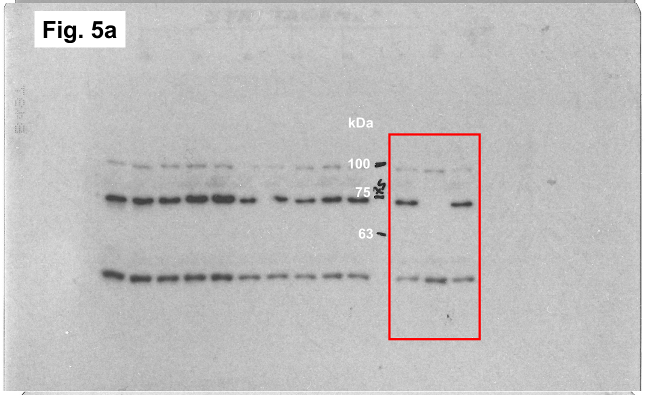

Fig. 5a. Middle image: Western anti-PBP2.

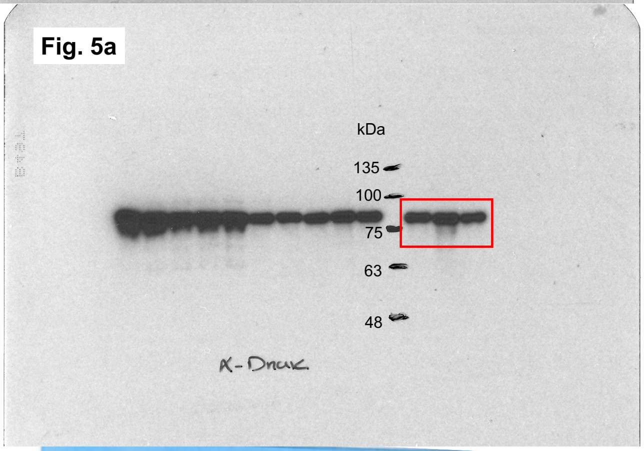

Fig. 5a. Lower image: Western anti-DnaK

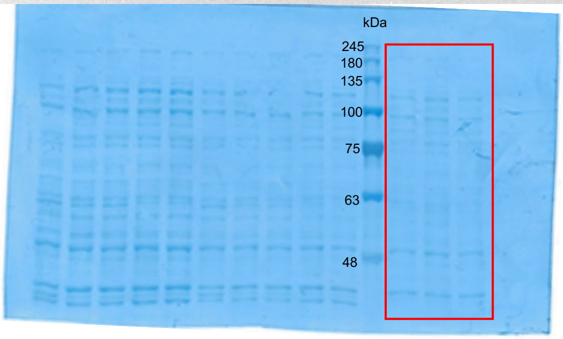

PVDF membrane of the blots shown in Fig. 5a

Supplementary Fig. 15. Original uncropped Western blots corresponding to Figure 5a of manuscript. Red boxes denote the areas of the blots that were used to compose Figure 5a.

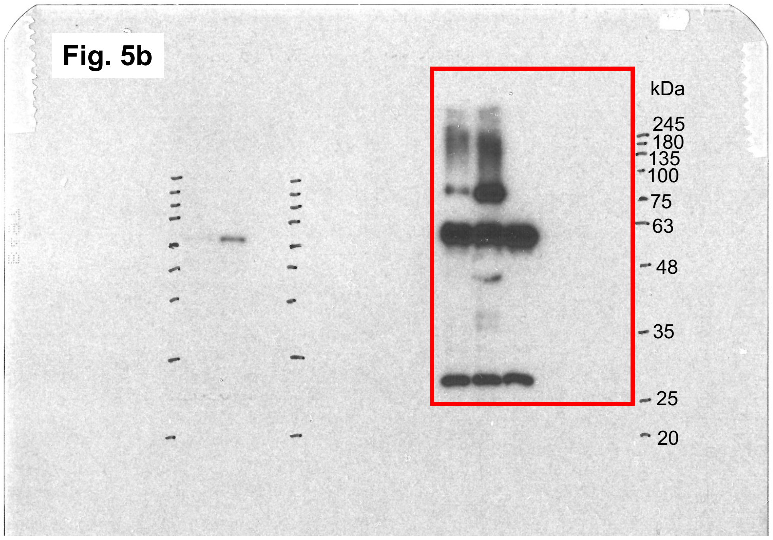

Fig. 5b. Left image: Western anti-FLAG

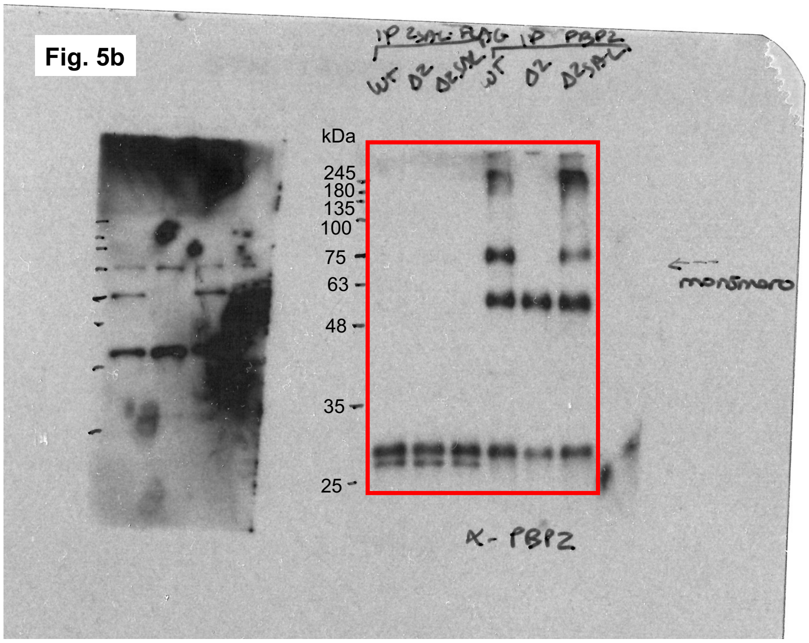

Fig. 5b. Right image: Western anti-PBP2

Supplementary Fig. 16. Original uncropped Western blots corresponding to Figure 5b of manuscript. Red boxes denote the areas of the blots that were used to compose Figure 5b.

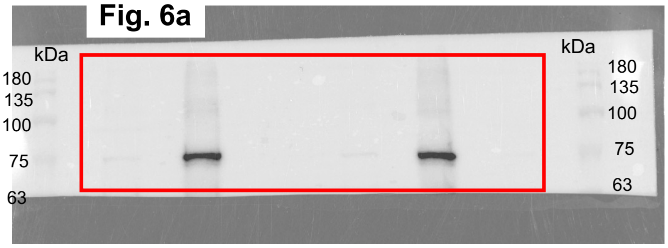

Fig. 6a. Upper image: Western anti-HA

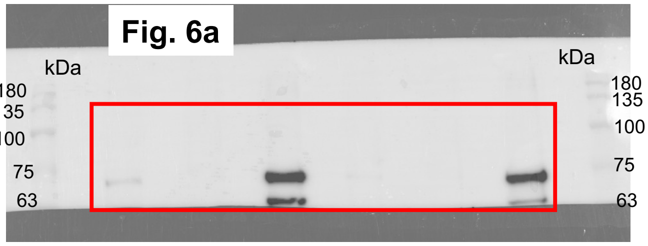

Fig. 6a. Middle-upper image: Western anti-PBP2

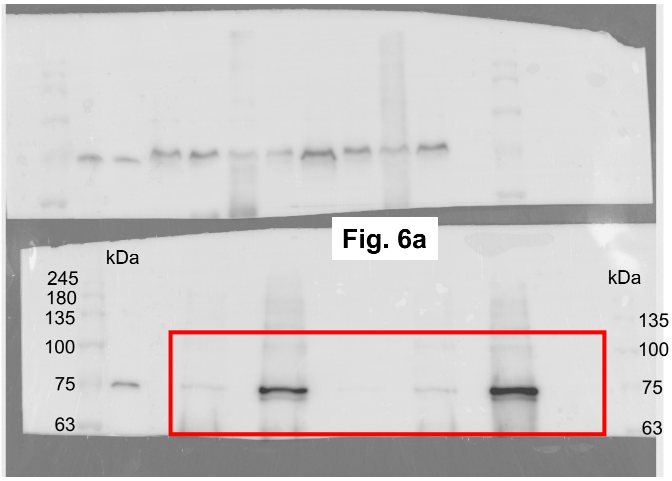

Fig. 6a. Middle-lower image: Western anti-HA

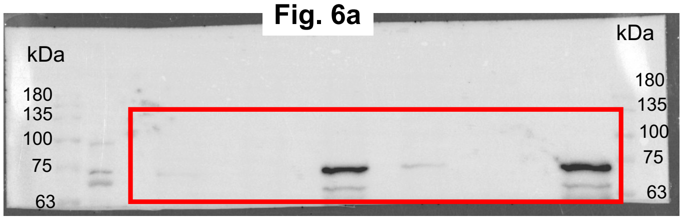

Fig. 6a. Lower image: Western anti-PBP2

Supplementary Fig. 17. Original uncropped Western blots corresponding to Figure 6a of manuscript. Red boxes denote the areas of the blots that were used to compose Figure 6a.

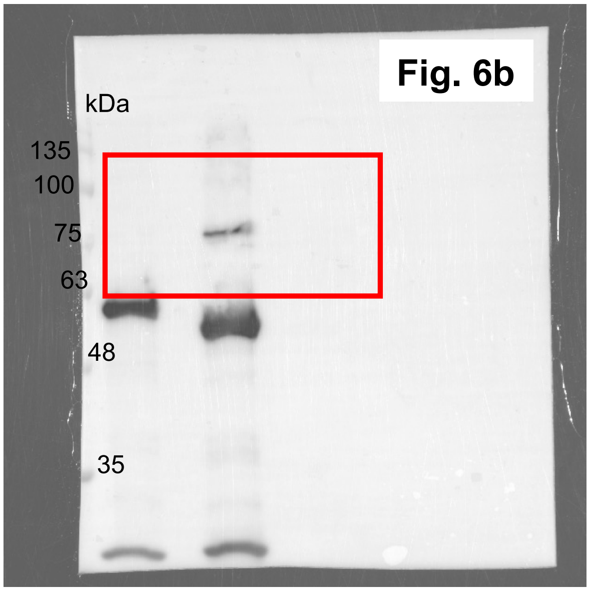

Fig. 6b. Upper image: Western anti-HA

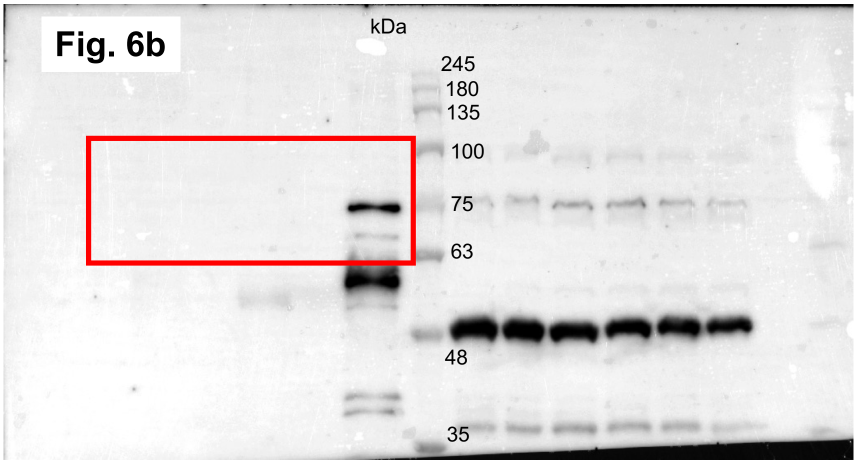

Fig. 6b. Lower image: Western anti-PBP2

Supplementary Fig. 18. Original uncropped Western blots corresponding to Figure 6b of manuscript. Red boxes denote the areas of the blots that were used to compose Figure 6b.

Fig. 6c. Upper image: Western anti-HA

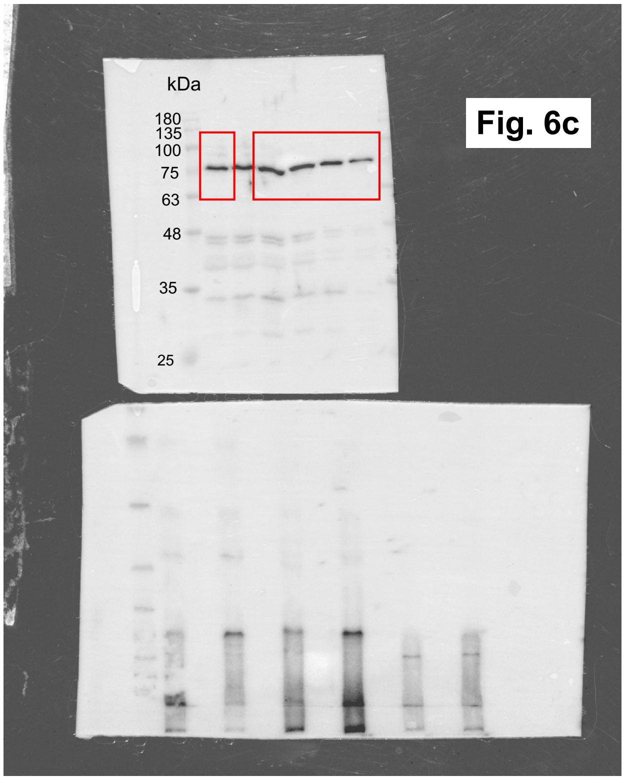

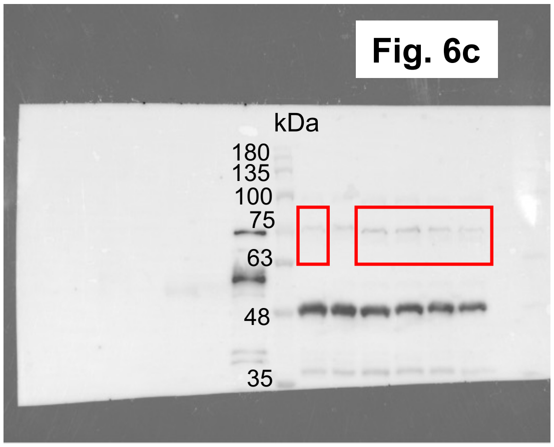

Fig. 6c. Middle image: Western anti-PBP2

Fig. 6c. Lower image: Western anti-FLAG

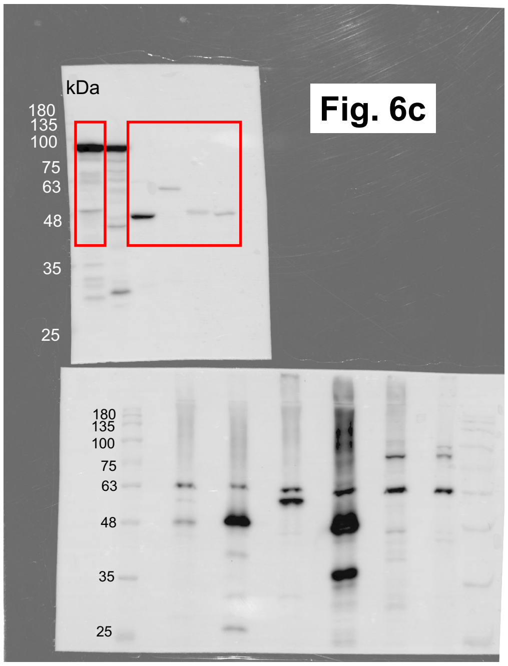

Supplementary Fig. 19. Original uncropped Western blots corresponding to Figure 6c of manuscript. Red boxes denote the areas of the blots that were used to compose Figure 6c.

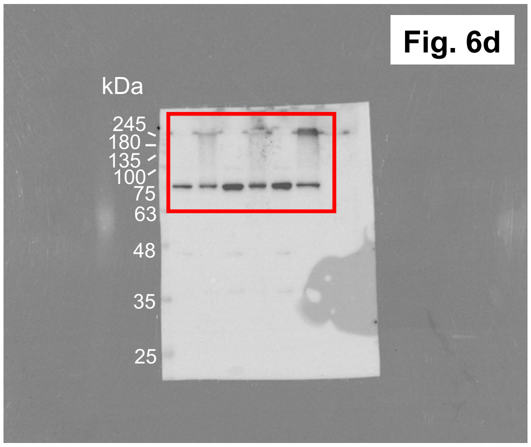

Fig. 6d. Left upper image: Western anti-HA

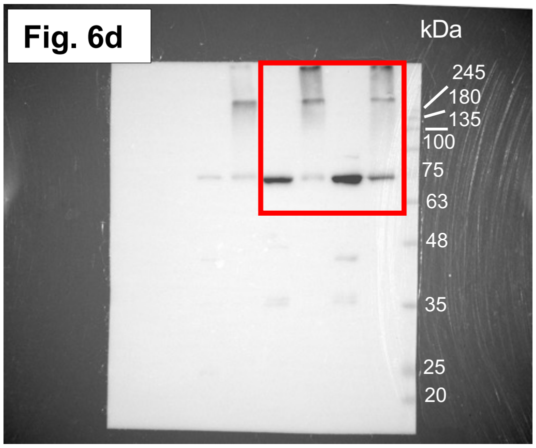

Fig. 6d. Right upper image: Western anti-HA

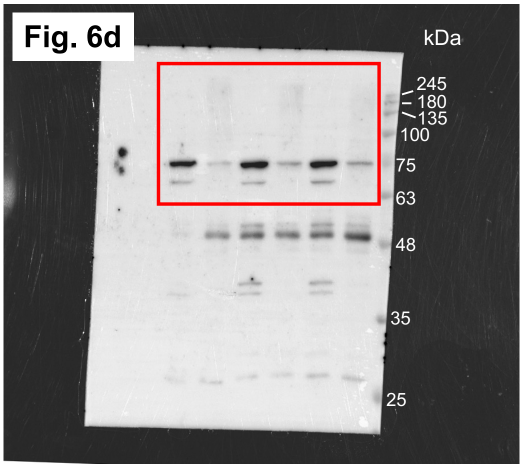

Fig. 6d. Left middle image: Western anti-PBP2

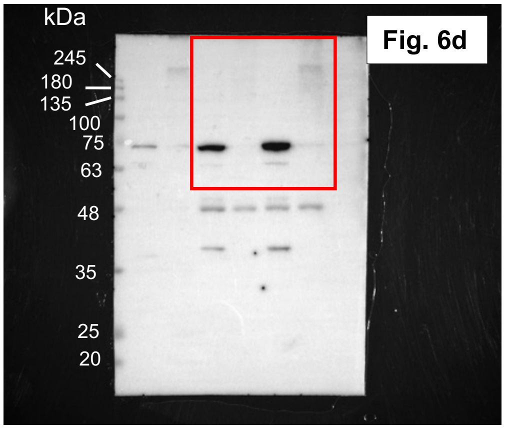

Fig. 6d. Right middle image: Western anti-PBP2

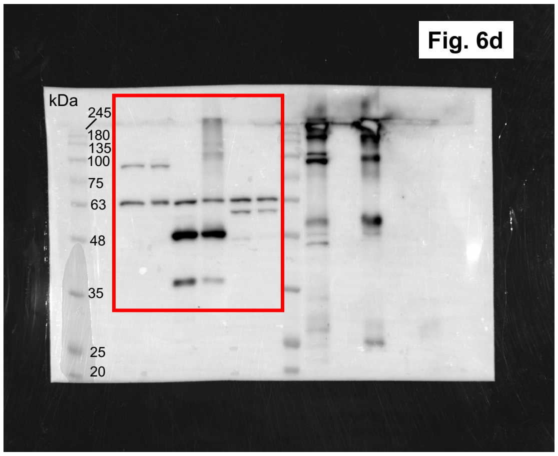

Fig. 6d. Left lower image: Western anti-FLAG

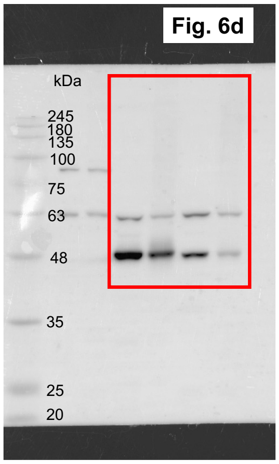

Fig. 6d. Right lower image: Western anti-FLAG

Supplementary Fig. 20. Original uncropped Western blots corresponding to Figure 6d of manuscript. Red boxes denote the areas of the blots that were used to compose Figure 6d.

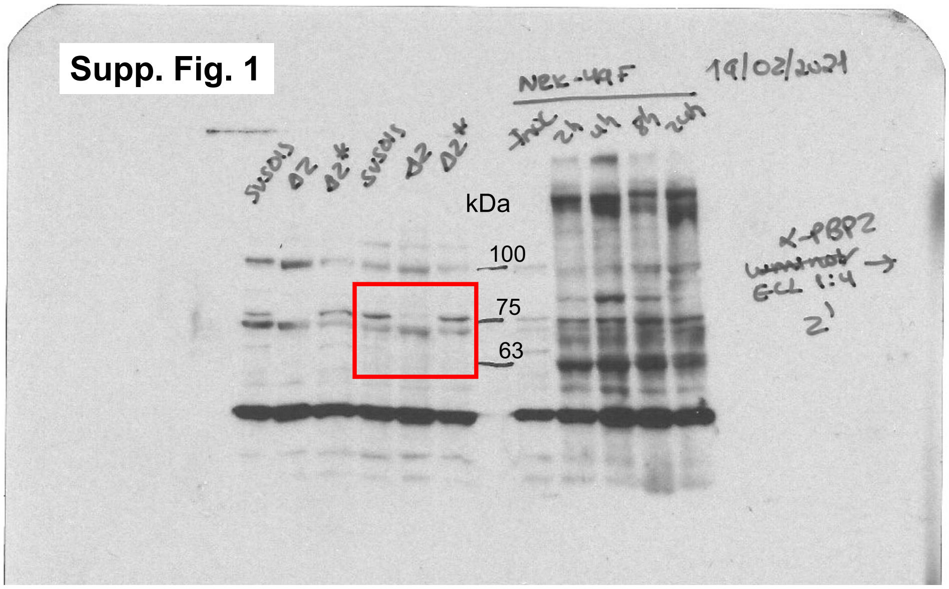

Supplementary Fig. 21. Image of original uncropped Western blot used in Supplementary Fig. 1. Red box denotes the area of the blots shown in Supplementary Fig. 1.

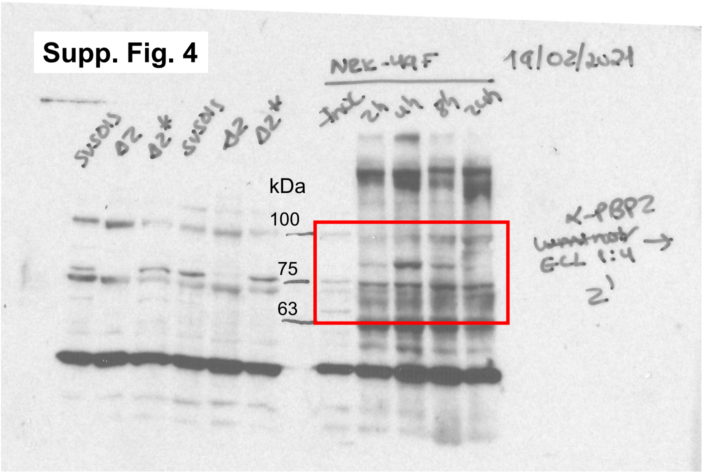

Supp. Fig. 4. Upper image: Western anti-PBP2

Supp. Fig. 4. Middle image: Western anti-FLAG

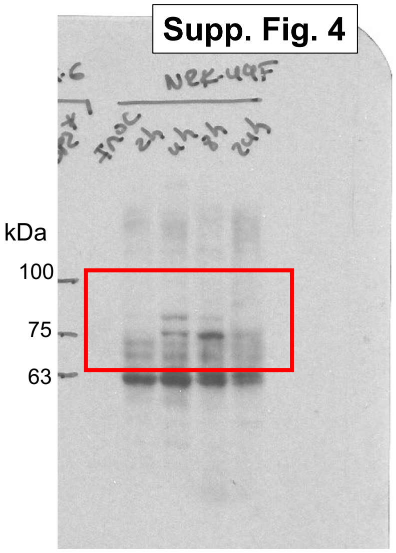

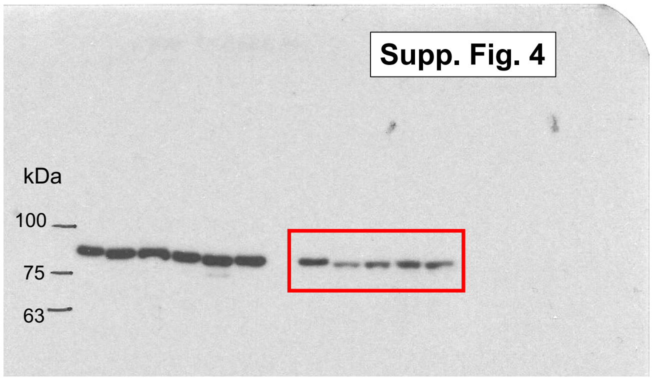

Supp. Fig. 4. Lower image: Western anti-DnaK

Supplementary Fig. 22. Images of original uncropped Western blots used in Supp. Fig. 4.

Red boxes denote the area of the blots shown in Supp. Fig. 4.

Supplementary Table 1. Single-nucleotide polymorphisms (SNPs) resulting in non-synonymous mutations identified by whole-genome sequencing (WGS) in the Δ*mrdA* null mutant described in this study, strain MD5052, derivate of strain SV5015.(*)

Coordinate SNP	Locus ID (gene)	Protein	Strain/ nucleotide change		Effect
			**SV5015** (†)	**MD5052** (Δ*mrdA*)	
635606	SL1344_0564 (*manX*)	PTS mannose transporter subunit IIAB transporter	A > T	A > T	Glu95Val
2135472	SL1344_2039 (*phsC*)	Thiosulfate reductase cytochrome B subunit	G > T	G > T	Gly110Val
2147535	SL1344_2048 (*hisG*)	ATP phosphoribosyl-transferase (HisG)	C > T	C > T	Pro69Leu
2409008	SL1344_2272	Hypothetical membrane protein	----	T > G	Val116Gly
2959813	SL1344_2772	Hypothetical membrane protein	----	T > G	Val405Gly
3994801	SL1344_3738	hypothetical NtrC-family transcriptional regulator	----	C > A	Gln261Lys

(*) WGS confirmed endpoints of the Δ*mrdA* deletion in coordinates 701512-703277. Genome comparisons were made against genome sequence of *S.* Typhimurium strain SL1344 (NCBI entry no. FQ312003.1).

(†) Strain SV5015 is the prototrophic His^+ strain used as wild type in this study. SV5015 was generated from the auxotrophic strain SL1344 by phage transduction and was also sequenced by WGS, as reported in our previous study[1].

Supplementary reference

1. Castanheira, S. *et al.* A Specialized Peptidoglycan Synthase Promotes Salmonella Cell Division inside Host Cells. *mBio* **8**, e01685-17 (2017).
