# Supplementary material for: Evidence of two differentially regulated elongasomes in Salmonella
Source: Commun Biol. 2023 Sep 9;6:923. doi: 10.1038/s42003-023-05308-w (PMC10492807; doi:10.1038/s42003-023-05308-w)
Supplement: Supplementary file 3 — Description of Additional Supplementary Data [file 42003_2023_5308_MOESM3_ESM.docx]

**Description of Additional Supplementary Files**

**File name:** Supplementary Data

**Description:** This file contains large tables in different tabs, each one referring to the corresponding figure of main paper or supplementary information.
