# Supplementary material for: Evidence of two differentially regulated elongasomes in Salmonella
Source: Commun Biol. 2023 Sep 9;6:923. doi: 10.1038/s42003-023-05308-w (PMC10492807; doi:10.1038/s42003-023-05308-w)
Supplement: Supplementary file 5 — Reporting-summary [file 42003_2023_5308_MOESM5_ESM.pdf]

nature portfolio

Corresponding author(s): García-del Portillo, Francisco

Last updated by author(s): Aug 17, 2023

Reporting Summary

Nature Portfolio wishes to improve the reproducibility of the work that we publish. This form provides structure for consistency and transparency in reporting. For further information on Nature Portfolio policies, see our Editorial Policies and the Editorial Policy Checklist.

Statistics

For all statistical analyses, confirm that the following items are present in the figure legend, table legend, main text, or Methods section.

n/a	Confirmed	
☐	☒	The exact sample size (*n*) for each experimental group/condition, given as a discrete number and unit of measurement
☐	☒	A statement on whether measurements were taken from distinct samples or whether the same sample was measured repeatedly
☐	☒	The statistical test(s) used AND whether they are one- or two-sided<br>*Only common tests should be described solely by name; describe more complex techniques in the Methods section.*
☒	☐	A description of all covariates tested
☒	☐	A description of any assumptions or corrections, such as tests of normality and adjustment for multiple comparisons
☐	☒	A full description of the statistical parameters including central tendency (e.g. means) or other basic estimates (e.g. regression coefficient) AND variation (e.g. standard deviation) or associated estimates of uncertainty (e.g. confidence intervals)
☐	☒	For null hypothesis testing, the test statistic (e.g. F, t, r) with confidence intervals, effect sizes, degrees of freedom and P value noted<br>*Give P values as exact values whenever suitable.*
☒	☐	For Bayesian analysis, information on the choice of priors and Markov chain Monte Carlo settings
☒	☐	For hierarchical and complex designs, identification of the appropriate level for tests and full reporting of outcomes
☒	☐	Estimates of effect sizes (e.g. Cohen's d, Pearson's r), indicating how they were calculated

Our web collection on statistics for biologists contains articles on many of the points above.

Software and code

Policy information about availability of computer code

Data collection	- Leica Application Suite (LAS) X (Leica software) v. 3.3.3.16958.<br>- NCBI (https://www.ncbi.nlm.nih.gov/) for collection of DNA sequences to perform phylogenetic analyses.
Data analysis	- Skyline v. 22.2 (https://skyline.ms/project/home/software/Skyline/begin.view)<br>- iTOL v. 6.5.7. (https://itol.embl.de/)<br>- Randomized Axelerated Maximum Likelihood (RAxML) v. 8.0 algorithm and LG amino acid replacement matrix integrated in the "Gene/Protein tree tool" of the Bacterial and Viral Bioinformatics Resource Center (BV-BRC) v. 2.29.20 (https://www.bv-brc.org/)<br>- Peaks v. 7.5 software as search engine in the proteomic analysis (https://www.bioinfor.com/).<br>- ObjectJ v.1.05n (as plug-in in Fiji -ImageJ2- v.2.9.0/1.53t)

For manuscripts utilizing custom algorithms or software that are central to the research but not yet described in published literature, software must be made available to editors and reviewers. We strongly encourage code deposition in a community repository (e.g. GitHub). See the Nature Portfolio guidelines for submitting code & software for further information.

Data

Policy information about availability of data

All manuscripts must include a data availability statement. This statement should provide the following information, where applicable:

- Accession codes, unique identifiers, or web links for publicly available datasets
- A description of any restrictions on data availability
- For clinical datasets or third party data, please ensure that the statement adheres to our policy

Source data are provided with this paper. The genome sequence of S. Typhimurium strain MD5052 (delta-mrdA) was uploaded to GenBank under BioProject ID PRJNA904137, Biosample accession SAMN31831537, SRA ID SRR22372222.
The mass spectrometry proteomics data have been deposited to the ProteomeXchange Consortium (https://www.proteomexchange.org/) via the PRIDE partner repository (https://www.ebi.ac.uk/pride/) with the dataset identifiers PXD039436 and 10.6019/PXD039436.
These datasets will be publicly available upon acceptance of the manuscript.

Human research participants

Policy information about studies involving human research participants and Sex and Gender in Research.

Reporting on sex and gender	Not applicable.
Population characteristics	Not applicable.
Recruitment	Not applicable.
Ethics oversight	Not applicable.

Note that full information on the approval of the study protocol must also be provided in the manuscript.

Field-specific reporting

Please select the one below that is the best fit for your research. If you are not sure, read the appropriate sections before making your selection.

☒ Life sciences ☐ Behavioural & social sciences ☐ Ecological, evolutionary & environmental sciences

For a reference copy of the document with all sections, see nature.com/documents/nr-reporting-summary-flat.pdf

Life sciences study design

All studies must disclose on these points even when the disclosure is negative.

Sample size	The determination of sample size for this study was not required for most of the experiments, which consisted on phenotypic analyses of wild type bacteria and mutants lacking defined PBPs. Only in the determination of length and width in bacterial populations, sizes were fixed to ranges of n≥100 for extracellular bacteria and n≥30 for intracellular bacteria.
Data exclusions	No data were excluded from the analyses.
Replication	The experiments were performed with a minimum of two independent biological replicates. Only exception was the proteomic analysis of the elongasome complex in bacteria grown in neutral pH.
Randomization	Randomization was not applicable in most assays since all bacterial strains used in the study harbor clearly-defined genetic differences. Nonetheless, microscopy images were analysed automatically in a randomized manner using tools as MicrobeJ and ObjectJ to obtain average morphogenetic parameters in bacterial populations.
Blinding	Blinding was not applicable since all assays involved comparisons among bacterial strains with clearly-defined genetic differences.

Reporting for specific materials, systems and methods

We require information from authors about some types of materials, experimental systems and methods used in many studies. Here, indicate whether each material, system or method listed is relevant to your study. If you are not sure if a list item applies to your research, read the appropriate section before selecting a response.

Materials & experimental systems

n/a	Involved in the study
☐	☒ Antibodies
☐	☒ Eukaryotic cell lines
☒	☐ Palaeontology and archaeology
☒	☐ Animals and other organisms
☒	☐ Clinical data
☒	☐ Dual use research of concern

Methods

n/a	Involved in the study
☒	☐ ChIP-seq
☒	☐ Flow cytometry
☒	☐ MRI-based neuroimaging

Antibodies

Antibodies used

- Polyclonal rabbit anti-O-antigen Salmonella (group B, factors 1,4,5,12) (cat. no. 229481) (Difco Antiserum BD Diagnostics, Sparks, MD)
- Affinity-purified polyclonal rabbit antibody anti-PBP2 of S. Typhimurium (lab collection, immune sera obtained from Charles River Laboratories)
- Affinity-purified polyclonal rabbit antibody anti-PBP3 of S. Typhimurium (lab collection, immune sera obtained from Charles River Laboratories)
- Mouse monoclonal anti-FLAG (cat. no. F3165) (Merck/Sigma-Aldrich)
- Mouse monoclonal anti-HA (cat. no. 901533) (BioLegend)
- Goat polyclonal anti-mouse IgG (H+L) conjugated to horseradish peroxidase (HRP) (cat. no. 1706516) (Bio-Rad)
- Goat polyclonal affinity-pure anti-rabbit IgG (H+L)-HRP conjugated (cat no. 1706515) (Bio-Rad)
- Goat polyclonal anti-rabbit IgG (H+L) alexa-fluor 488 conjugated (cat no. A-11008) (ThermoFisher Scientific)

Validation

Primary antibodies were validated with samples obtained from bacteria lacking the genes encoding the corresponding proteins (PBP2 or PBP3), which served as negative controls in the Western blots. The anti-O-antigen of Salmonella was validated with slide agglutination tests.

Eukaryotic cell lines

Policy information about cell lines and Sex and Gender in Research

Cell line source(s)	NRK-49F rat kidney fibroblasts (American Type Culture Collection, ATCC-1570)
Authentication	Cell line received directly from ATCC. Authenticated by the supplier.
Mycoplasma contamination	Cell line tested for Mycoplasma contamination upon receiving cell line from ATCC with negative results.
Commonly misidentified lines (See ICLAC register)	No misidentified cell lines were used.
